# Designation of a neotype for *Enteromiuspallidus* (Smith, 1841), an endemic cyprinid minnow from the Cape Fold Ecoregion, South Africa

**DOI:** 10.3897/zookeys.848.32211

**Published:** 2019-05-20

**Authors:** Melissa B. Martin, Albert Chakona

**Affiliations:** 1 School of Marine and Environmental Sciences, Universiti Malaysia Terengganu, Kuala Nerus, Terengganu Darul Iman, 21030, Malaysia Universiti Malaysia Terengganu Kuala Nerus Malaysia; 2 South African Institute for Aquatic Biodiversity, Private Bag 1015, Grahamstown, 6140, South Africa South African Institute for Aquatic Biodiversity Grahamstown South Africa

**Keywords:** *
Enteromius
*, freshwater fish, Baakens River, Eastern Cape Province, southern Africa

## Abstract

*Enteromiuspallidus* was described by Smith in 1841 without a designated type specimen for the species. Herein, we designate a specimen from the Baakens River system as a neotype for *E.pallidus* and provide a thorough description for this species to facilitate ongoing taxonomic revisions of southern African *Enteromius*. *Enteromiuspallidus* can be distinguished from the other minnows in the “goldie barb group” by having an incomplete lateral line, lack of distinct chevron or tubular markings around lateral line pores, absence of a distinct lateral stripe, absence of wavy parallel lines along scale rows and lack of black pigmentation around the borders of the scales. We provide mtDNA COI sequences for the neotype and an additional specimen from the Baakens River as DNA barcodes of types and topotypes are a fundamental requirement for further taxonomic studies.

## Introduction

The Cyprinidae is one of the most widespread and species-rich freshwater fish families, with 1685 valid species worldwide (Eschmeyer et al. 2018). The African continent currently contains at least 475 species in 24 genera, with the Congo River system being the centre of cyprinid diversity (Eschmeyer et al. 2018). The African cyprinids can be broadly divided into the small diploid species (e.g. *Caecobarbus*, *Barbopsis*, *Clypeobarbus*, *Barboides* and species that were previously referred to as *Barbus* or ‘*Barbus*’), small-to-medium sized tetraploid species (e.g. *Pseudobarbus*) and the large-sized hexaploid species (e.g. *Labeobarbus*) (Agnèse et al. 1990; Berrebi et al. 1996; Berrebi and Valiushok 1998; Ren and Mayden 2016; Van Ginneken et al. 2017). Recently, Yang et al. (2015) proposed that the small-sized African diploid minnows that were previously variously referred to as either *Barbus* or ‘*Barbus*’ (Berrebi et al. 1996) should be preliminarily combined under the name *Enteromius* Cope, 1867 in the tribe Smiliogastrini. This suggestion has been provisionally accepted, pending a critical evaluation of the generic status of the African diploid minnows (e.g. Skelton 2016; Hayes and Armbruster 2017; Van Ginneken et al. 2017; Schmidt et al. 2017, 2018).

*Enteromius* is currently represented by 350 valid species, making it the most speciose and widely distributed cyprinid genus on the African continent (Hayes and Armbruster 2017), and new species have been recently described (e.g. Lederoun and Vreven 2016), revalidated (e.g. Schmidt et al. 2018) or await formal description (Van Ginneken et al. 2017). The genus *Enteromius* is distinguished from other small African diploid smiliogastrin genera (*Barboides*, *Barbopsis*, *Caecobarbus*, *Clypeobarbus*) based on differences in dorsal-fin placement in comparison to anal-fin origin, number of dorsal-fin rays, number of paired nostrils on either side of the snout, eye size, placement in the orbital rim and pigmentation pattern, shape and pattern of midlateral scale row (Hayes and Armbruster 2017). In southern Africa, this genus is represented by 38 species (Skelton 2001).

Despite *Enteromius* being the most common genus occurring in almost all river systems across the continent, these fishes are generally difficult to identify because of their very similar body morphology and colour pattern, coupled with the lack of revision within the group (Hayes and Armbruster 2017; Van Ginneken et al. 2017). As a result, a number of species within *Enteromius* are currently considered to have wide geographic ranges across multiple river systems (Skelton 2001). Such distribution patterns are unexpected for freshwater restricted taxa as their dispersal is limited by terrestrial and marine barriers, and they reflect the incomplete systematic and taxonomic knowledge of freshwater fishes in the region. This “taxonomic impediment” handicaps basic research in biological sciences and biodiversity conservation.

**Figure 1.**
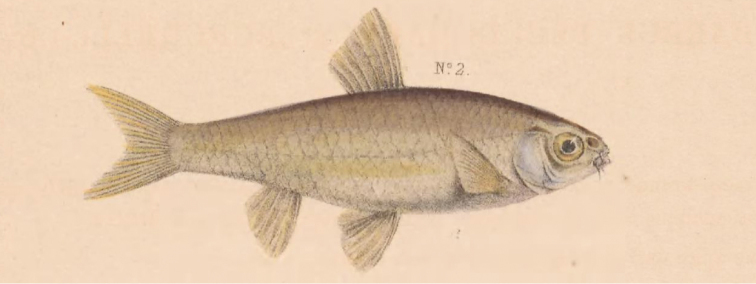
Illustration of *Enteromiuspallidus* [formerly Barbus (Pseudobarbus) pallidus] from Smith (1841).

**Figure 2.**
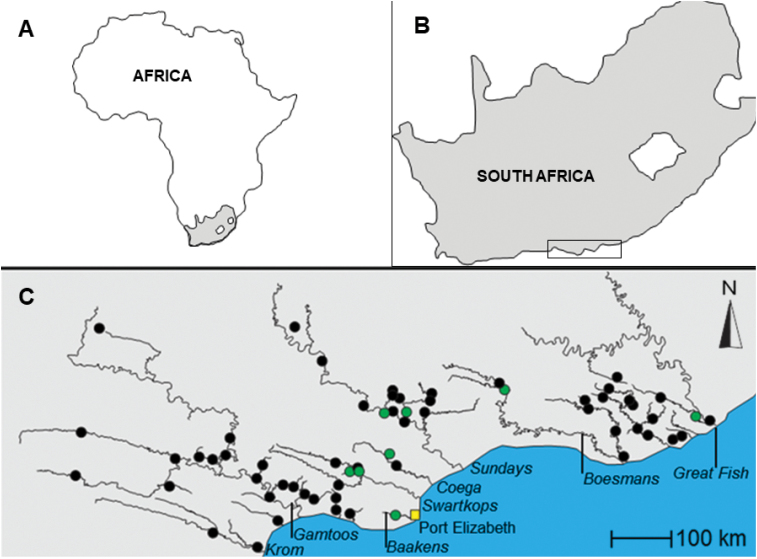
Map of the African continent (**A**) showing the position of South Africa (**B**), and the distribution of *Enteromiuspallidus* in the eastern Cape Fold freshwater ecoregion (**C**). The Baakens River, which is the type locality of *E.pallidus*, is now entirely contained within the city of Port Elizabeth (Nelson Mandela Metropolitan). Green dots represent sampling localities for the tissue samples that were used for the genetic study of Chakona et al. (2015).

The present study forms part of an ongoing comprehensive taxonomic revision of the goldie barb group which comprises three cyprinid minnows endemic to southern Africa, *E.pallidus*, *E.brevipinnis* (Jubb, 1966) and *E.neefi* (Greenwood, 1962). There are however no existing type specimens for *E.pallidus* (Eschmeyer et al., 2018). *Enteromiuspallidus* (Fig. 1) was described by Smith (1838–47) as Barbus (Pseudobarbus) pallidus, with the type locality listed as ‘various parts of Cape Colony’. This potentially encompassed any of the southern coastal river systems in the present-day Eastern Cape Province of South Africa, from the Krom to the Great Fish, where *E.pallidus* is known to occur (Skelton 2001). The likely type locality is the Baakens River in Port Elizabeth (Fig. 2) as that is close to Fort Frederick, the former British Military establishment in the town, where Andrew Smith, who was a British army surgeon, might have been based between 1821–1825 when he was posted to the eastern frontier and at other times after he moved to Cape Town. Boulenger (1911) described *Barbushemipleurogramma* from the Baakens River, but Barnard (1943) put this species into synonymy with *B.pallidus*.

The name *E.pallidus* (previously *B.pallidus*) has been applied for minnows with scattered spots on the lateral and dorsal side of the body from other river systems in South Africa, including tributaries of the Orange-Vaal, Tugela, Mfolozi, Pongolo, Incomati and Limpopo river systems. The species has, thus, for a long time been considered to have a distribution pattern divided into coastal and inland populations (Skelton 2001). Most recently, Chakona et al. (2015) revealed substantial genetic differentiation between the coastal and inland populations of *E.pallidus* and showed that the inland lineage is not closely related to *E.pallidus* s.s. As there are no types for *E.pallidus*, the aim of the present study was to designate a neotype and provide detailed description of this species based on the topotypic specimens collected from the Baakens River system in Port Elizabeth. The present study thus provides clarity on the likely type locality of *E.pallidus* and presents an accurate definition for this species in accordance with Article 75.3.1 of the International Code for Zoological Nomenclature, ICZN (International Commission on Zoological Nomenclature 1999). This is a fundamental requirement for future taxonomic comparisons and revision of spotted smiliogastrins in southern Africa whose taxonomic status is currently uncertain.

## Materials and methods

### Sample collection and deposition

Fishes were collected on the 3^rd^ November 2018 using a seine net (3 m long, 3 mm mesh size). Captured fishes were anaesthetised with clove oil (0.2%) and digitally photographed using a Nikon D3100 7.4/9V camera on site to capture live colour pattern. For genetic analysis, a small piece of muscle tissue was dissected from the right side of each specimen in the field, preserved in 95% ethanol and later stored at -20°C in the molecular laboratory at the South African Institute for Aquatic Biodiversity (SAIAB). Voucher specimens were fixed in 10% formalin in the field. They were then put through 10% and 50% ethanol washes to rinse the formalin and eventually transferred to 70% ethanol for long-term storage. The neotype (SAIAB 207086) and additional topotypes (SAIAB 207084) were deposited into the fish collection facility at SAIAB as reference material. Permission for sampling was obtained from the Department of Economic Development, Environmental Affairs and Tourism (Eastern Cape Province) (permit number: CRO 44/18CR).

### Morphological analyses

Meristic and morphological characters were selected as defined by Hubbs and Lagler (1958), Skelton (1988), Chakona and Swartz (2013) and Chakona et al. (2014). Morphometric measurements were taken point-to-point using an IP54 digital caliper to 0.1 mm precision. The characters considered for each specimen in the present study (22 morphometric measurements and 16 meristic counts) are presented in Chakona et al. (2014).

### Molecular data

We provide mtDNA COI barcode sequences for the neotype (designated as neogenetype) and an additional specimen (designated as topogenetype) following definitions of Chakrabarty (2010) as these sequences will facilitate detailed phylogenetic analyses to determine the relationships of *E.pallidus* and other southern African congeners as more data become available through ongoing studies. These sequences were deposited in GenBank: neogenetype (MK900662) and topogenetype (MK900663). DNA extraction, PCR and sequencing methods follow Chakona et al. (2018).

## Results

### Enteromius pallidus

Taxon classificationAnimaliaCypriniformesCyprinidae

(Smith, 1841)

Figs 3 , 4

Barbus (Pseudobarbus) pallidus Smith, 1841: no pagination, pl. 11 (fig. 2). Type locality: Defined in the original description as “various parts of the Cape Colony”, but it is likely to be the Baakens River which is closest to the former British Army base, Fort Fredrick, where Andrew Smith, who was an army surgeon, would have been based at the time when he described this species.
Barbus hemipleurogramma Boulenger, 1911, fig. 126. Type locality: Baakens River, Port Elizabeth, Cape Province, South Africa; Bertin and Estève 1948.
Barbus pallidus : Barnard 1943; Lévêque et al. 1984; Skelton 1993; Engelbrecht and van der Bank 1996; Seegers 1996, Farm 2000; Skelton 2001; Muller et al. 2015; Chakona et al. 2015.
Enteromius pallidus : Hayes and Armbruster 2017.

#### Material examined.

**Figure 3.**
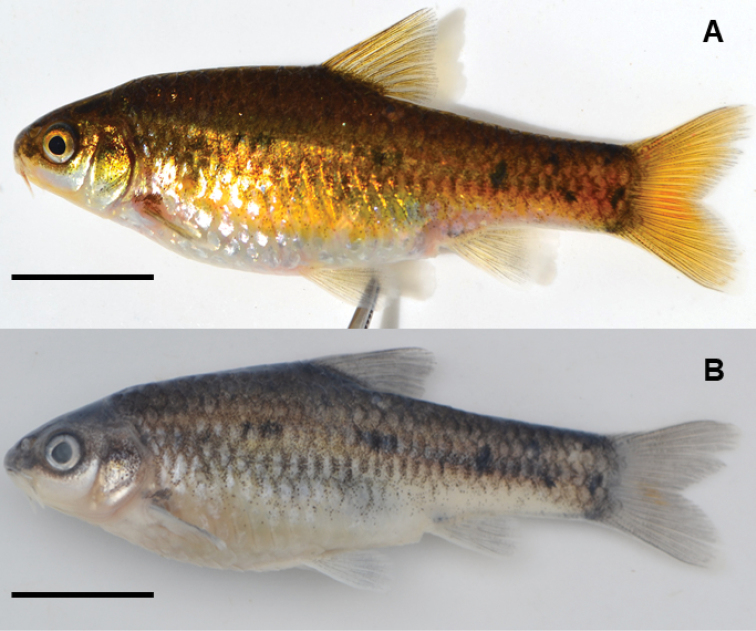
General body features and live (**A**) and alcohol preserved (**B**) coloration of the neotype of *Enteromiuspallidus* (SAIAB 207086), a gravid adult female. Scale bar: 10 mm.

**Neotype** (Fig. 3A, B): In compliance with Article 75.3.7, the neotype was deposited at the South African Institute for Aquatic Biodiversity (catalogue no. SAIAB 207086) for future reference. The neotype is an adult female, 51.4 mm standard length (SL), collected on 3 November 2018 by Albert Chakona, Wilbert Kadye and Melissa Martin using seine netting, Baakens River system at Targetklooff downstream of bridge on the road to Walmer, (33°58'12"S, 25°35'40"E), altitude 20 m, Port Elizabeth, South Africa.

**Figure 4.**
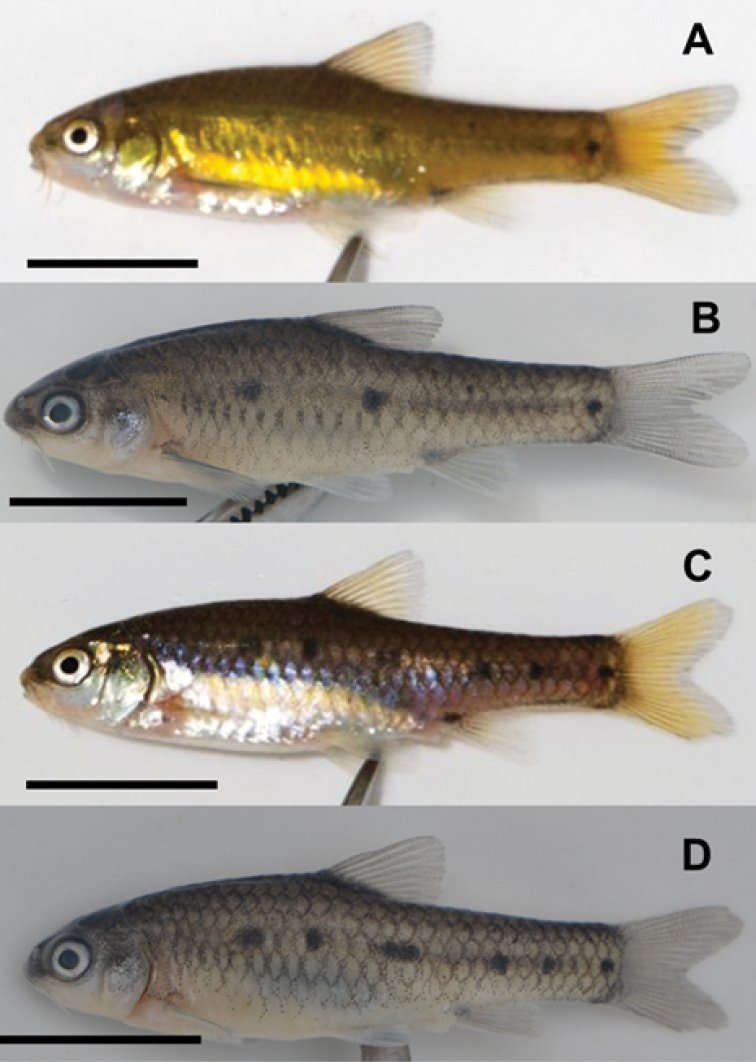
General body features and live (**A**) and alcohol preserved (**B**) coloration of a mature breeding male *E.pallidus* (**A, B**SAIAB 207084), and live (**C**) and alcohol preserved (**D**) coloration of an unsexed sub-adult (SAIAB 207084). Scale bar: 10 mm.

**Additional material.** South Africa: Port Elizabeth: SAIAB 207084, (*n*= 6; 2 adult females, 1 adult male, 4 sub-adults), 17.1–36.1 mm standard length (SL), collection details similar to neotype (Fig. 4A–D).

South Africa: Eastern Cape: Port Elizabeth: SAIAB 200091; (n=38 juveniles and sub-adults, 17.1–36.1 mm standard length (SL), collected from the Baakens river on 4^th^ April 2014 by Albert Chakona and Roger Bills downstream of low water bridge on the road to Green Acres, (33°57'28.1"S, 25°33'36.8"E).

South Africa: Eastern Cape: Port Elizabeth: SAIAB 127772; (n=2 gravid females, 46.8 and 47.6 mm standard length (SL), collected on 22 October 1981 by D. Heard from the Baakens River system, (33°58'S, 25°37'E).

#### Neotype designation for *Enteromiuspallidus* (Smith, 1841).

The generic status of the diploid Smiliogastrini minnows, currently placed in *Enteromius*, is the subject of ongoing investigation because this genus is polyphyletic (Yang et al. 2015, Ren and Mayden 2016, Hayes and Armbruster 2017). Detailed revision of the taxonomic statuses of the species belonging to this genus is plagued by a number of challenges, particularly similar body morphology, ambiguous type locality details and lack of extant type material for a number of species. This hinders accurate resolution of species identities, resulting in exaggerated geographic distribution ranges for many of the species (see Skelton, 2001). Without primary type specimens and better resolution of species identities and their distribution ranges, it would be difficult to resolve the bigger questions of the generic status and relationships of diploid smiliogastrins. Within southern Africa, a number of species in the genus *Enteromius* are in need of taxonomic revision as many are perceived to have broad geographic ranges, such as the case of *E.pallidus*. There are no extant types for this species, the illustration does not provide clear diagnostic characters to objectively associate it with *E.pallidus* or for comparisons with other species, and the species has a vague type locality description. The designation of a neotype is therefore essential to facilitate ongoing taxonomic revision of the “goldie barb” complex in southern Africa and for the broader evaluation of the phylogenetic relationships and generic placements of *Enteromius* species across the African continent. There is therefore an explicit need for the designation of a neotype (Art. 75.3 of ICZN).

**All qualifying conditions (Art. 75.3 of ICZN) are met.** The neotype is designated to clarify the taxonomic status of the species (Art. 75.3.1). *Enteromiuspallidus* was described by Smith, who provided an illustration for a specimen with a brief description of the colour and form of the species, and a vague type locality defined as “clear streams in various parts of the Cape colony”. Although Smith provided an illustration, there is no evidence within the text that he established a holotype or any expression of the equivalent. In compliance with Article 75.3.4 of the ICZN, the authors conducted a comprehensive search for the types, and it was established that extant types for *E.pallidus* are unlikely to be in existence. This was based on correspondences with Prof. Paul Skelton at the South African Institute for Aquatic Biodiversity (SAIAB), who confirmed that he examined and measured all the types of southern African freshwater fishes in 1981 at the British Museum of Natural History (BMNH). He searched for Smith’s *Barbuspallidus* but found no trace of any record or specimen(s). The authors also contacted the curator at the British Museum, James Maclaine, who indicated that Andrew Smith’s types of *E.pallidus* are not at the BMNH. While according to Article 73.1.4, Smith’s (1841) illustration would be considered to represent the holotype of *E.pallidus*, unfortunately the illustration does not provide clear details to extract diagnostic features for the species.

In compliance with Articles 75.3.2 and 75.3.3, a diagnosis, redescription, and comparison of *E.pallidus* and the other congeners in southern Africa are presented below. Following Barnard (1943), the original specimens used for the description of *E.pallidus* could have come from a river system near Port Elizabeth, probably the Baakens River. We therefore chose a specimen from the Baakens River system for the neotype designation (in compliance with Article 75.3.6), because it is closest to the 1820’s British army camp (Fort Frederick), where Andrew Smith is likely to have been based during the time when he made the description.

#### Diagnosis.

*Enteromiuspallidus* can be identified by the slightly convex dorsal surface; posterior barbel 2.0 to 3.0 times the length of anterior barbel; a slightly prominent snout; an incomplete lateral line; deep translucent light brown to golden sheen with the presence of irregular and scattered spots in mature adults; and the presence of 3–7 bold spots above the lateral line in juveniles and sub-adults.

#### Comparison with congeners in southern Africa.

The species belongs to the group of *Enteromius* species in southern Africa that is characterised by a simple and flexible unbranched primary dorsal fin ray. Distinguished from *E.amatolicus* (Skelton, 1990), *E.anoplus* (Weber, 1897), *E.annectens* (Gilchrist & Thompson, 1917), *E.toppini* (Boulenger, 1916) and *E.radiatus* (Peters, 1853) by possession of two pairs of prominent and long barbels (*vs* single pair and/or minute oral barbels in other species). Distinguished from *E.lineomaculatus* (Boulenger, 1903), *E.viviparus* (Weber, 1897) and *E.unitaeniatus* (Günther, 1867) by absence of distinct chevron markings on the lateral line (*vs* presence of conspicuous chevron markings on the lateral line in the other three species), and from *E.bifrenatus* (Fowler, 1935) by absence of a distinct lateral stripe and absence of black tubular markings around lateral line pores (*vs* presence in *E.bifrenatus*). Distinguished from *E.anoplus*, *E.amatolicus*, *E.annectens*, *E.unitaeniatus*, *E.bifrenatus*, *E.gurneyi* (Günther, 1868), *E.motebensis* (Steindachner, 1894), *E.radiatus*, *E.toppini*, *E.treurensis* (Groenewald, 1958) and *E.viviparus* by the presence of scattered black spots on the body, particularly in juveniles (*vs* absence of scattered black spots in the other species). Lateral pigmentation pattern of *E.pallidus* is closely similar to that of *E.brevipinnis* and *E.neefi* (Greenwood, 1962), but it is distinguished from these two species by having an incomplete lateral line (*vs* complete lateral line in both *E.neefi* and *E.brevipinnis*). *Enteromiuspallidus* is further separated from *E.neefi* by absence of wavy lines along the scale rows (*vs.* presence of conspicuous wavy lines along the scale rows in *E.neefi*), and from *E.brevipinnis* by lack of black pigmentation around the borders of the scales (*vs* presence of distinct black pigmentation around the scales in *E.brevipinnis*, giving a mesh-like pattern on the lateral side of the fish).

Figures 3, 4 show the general body features of *E.pallidus* as an adult female (neotype), adult male and juvenile. Morphometric and meristic data for the neotype and additional (topotypic) material are presented in Table 1.

**Table 1.** Morphometric measurements and meristic counts of *Enteromiuspallidus* neotype and additional material from Baakens River. Ranges of characters are presented first, followed by the mean and standard deviation in parentheses. Meristic characters are given in the range first, with the mode in parentheses.

	* Enteromius pallidus *	
	Neotype	Additional material
No. of specimens	n=1	n=46
Morphometrics (mm)		
Standard length (SL) (mm)	51.4	17.1–49.3 (26.8; 8.1)
Head length (HL) (mm)	9.4	3.8–10.7 (5.7; 1.6)
Percentage of SL (%)		
Head length	18.3	17.9–25.1 (21.5; 1.6)
Predorsal length	54.1	46.9–56.2 (53.1; 1.9)
Dorsal fin base	10.5	4.7–20.3 (10.6; 2.8)
Dorsal fin height	20.8	16.5–27.0 (21.3; 2.3)
Body depth	29.9	20.5–30.9 (26.2; 1.9)
Body width	16.9	7.3–20.4 (11.4; 2.5)
Caudal peduncle length	20.4	19.9–32.8 (27.8; 2.9)
Preanal length	69.2	59.8–73.7 (68.7; 2.9)
Prepelvic length	47.7	42.7–54.3 (49.2; 2.5)
Pelvic fin length	13.0	12.6–21.2 (16.1; 1.5)
Pectoral to pelvic fin length	22.8	16.3–28.2 (21.2; 2.5)
Pelvic to anal fin length	17.9	12.2–21.6 (17.3; 1.9)
Anal fin base	7.59	2.9–9.0 (6.3; 1.3)
Percentage of HL (%)		
Head depth	105.3	75.5–109.0 (92.2; 7.5)
Snout length	31.9	20.0–44.4 (33.6; 5.5)
Orbit diameter	36.2	31.4–51.2 (40.5; 5.3)
Postorbital length	54.3	40.8–67.2 (55.2; 5.3)
Interorbital width	57.4	44.2–66.7 (55.7; 6.3)
Anterior barbel length	16.0	4.1–30.4 (15.7; 7.3)
Posterior barbel length	30.9	21.7–64.1 (37.5; 10.5)
Percentage of caudal peduncle length (%)		
Caudal peduncle depth	13.2	10.1–15.0 (12.6; 1.0)
Meristics		
Unbranched dorsal fin rays	3	3(3)
Branched dorsal fin rays	7	7 (7)
Unbranched anal fin rays	3	3 (3)
Branched anal fin rays	5	5 (5)
Unbranched pectoral fin rays	1	1 (1)
Branched pectoral fin rays	7	7 (7)
Unbranched pelvic fin rays	1	1 (1)
Branched pelvic fin rays	7	5–7 (5)
Unbranched caudal fin rays	2	2 (2)
Branched caudal fin rays	17	15–19 (17)
Lateral line scales	13	5–19 (9)
Number of scales in lateral series	31	23–30 (26)
Scales between lateral line and dorsal fin origin	4	3–5 (4)
Scales between lateral line and pelvic fin origin	2–3	2–5 (3)
Scales between lateral line and anal fin origin	2	2–3 (2)
Circumpeduncular scales	12	12 (12)
Predorsal scale rows	10	7–14 (10)

#### Neotype description

(Article 75.3.3.). (Fig. 3A, B). *Body* fusiform, moderately compressed laterally; with four visible, irregular spots above lateral line. *Dorsal profile* slightly convex from tip of snout to origin of dorsal fin; anterior-projection slightly pronounced; body depth greatest between dorsal fin and anal fin origin, tapering from posterior margin of dorsal fin base to base of caudal fin. *Ventral profile* slightly concave, curving downwards from operculum to origin of pelvic fin base, slightly tapering to posterior end of anal fin base, then slightly concave to caudal fin.

*Head* relatively small and slightly projected; 0.2 times standard length, head length sub-equal to body depth. *Eye* relatively large and round; located dorsolaterally, closer to tip of snout than distal margin of operculum, interorbital space slightly convex. *Snout* rounded, shorter than post-orbital length; sub-equal or less than eye diameter; nuptial tubercles absent.

*Mouth* inferior; upper jaw sub-equal to lower jaw. *Lip* simple and thin; lower lip unretracted. *Two pairs of barbels*; rostral (anterior) barbels minute, reaching past posterior end of nostril, 0.3 times length of eye diameter; *maxillary* (posterior) *barbels* 3.0 times longer than rostral barbels, reaching beyond vertical through middle of eye.

*Dorsal fin* with 3 simple unbranched and 7 branched rays; distal margin almost straight; origin centered vertically with origin of pelvic fins. *Pectoral fin* with 1 simple unbranched and 7 branched rays; posterior edge gently rounded, not reaching pelvic fin origin. *Pelvic fin* with 1 simple unbranched and 5 branched rays; posterior edge gently rounded, almost reaching anus; origin midway between pectoral fin origin and anal fin origin. *Anal fin* with 3 unbranched and 5 branched rays; distal margin almost straight; origin inserted closer to origin of pelvic fin than base of caudal fin. *Caudal fin* bifurcate; with two pairs of 1 simple unbranched ray, 8 or 9 branched rays on each lobe.

*Scales* moderately large, radiately striated. *Lateral line* incomplete, with 4–13 (mode 9) perforated scales, 23–31 (mode 26) lateral scale series; 3–5 (mode 4) scale rows between dorsal fin origin and lateral line; 2–5 (mode 3) scale rows between pelvic fin origin and lateral line; 2–3 (mode 2) scale rows between lateral line and anal fin origin; 12 circumpeduncular scale rows; 7–14 (mode 10) predorsal scale rows, embedded in skin, smaller than flank scales. Scales between posterior base of pectoral fins and anterior base of pelvic fins smaller than flank scales and embedded.

#### Coloration.

In life, the colour for both adult breeding males and females is deep greenish-brown with a golden sheen dorsally, golden-yellow laterally and silvery ventrally (Figs 3A, B; 4A, B). Fins are translucent-yellow. The neotype thus represents *E.pallidus* sensu Smith (1841) based on the consistent similarities in colour pattern as defined in the original description (Art. 75.3.5). Juveniles appear brown laterally and silvery ventrally. Black spots are present above the lateral line, with juveniles and sub-adults having bold or more prominent spots in comparison to adults which tend to have fewer and often less conspicuous spots or blotches. All the juveniles and sub-adults examined (46 in total) had at least 3 bold spots above the lateral line (4C and 4D) on both sides (range 3–7 bold lateral spots). At least one bold spot is consistently found within the pre-dorsal region, pre-anal and caudal regions, a dark spot is always present on vertical through dorsal fin insertion and at the base of the caudal peduncle. Alcohol preserved specimens appear either plain silvery, or dusky grey dorsally and laterally and cream-yellowish ventrally (Figs 3B; 4B, D). The black spots become more prominent in preserved specimens. Black pigmentation at the anterior base of the anal fin is more prominent in juveniles and sub-adults compared to adults.

#### Reproduction.

There have been no dedicated studies on the breeding biology of *E.pallidus*, but spawning is likely to begin in summer (October – November) based on the general pattern of other congeners (Cambray and Bruton 1984; Skelton 2001), and other cyprinid minnows in the CFE (Cambray 1994). We have also observed presence of several gravid females and males with breeding coloration (prominent golden-yellowish sheen) during field surveys conducted during the summer period.

#### Distribution and habitat.

*Enteromiuspallidus* is endemic to the eastern Cape Fold Ecoregion (CFE) of South Africa where it is distributed from the Krom to the Great Fish river system (Fig. 2). Rivers in this region are characterized by variable flow regimes, with mountain tributaries generally flowing throughout the year, while some main-stem sections of the rivers recede into a series of disconnected pools during the dry season (O’Keeffe and de Moor 1988). The species inhabits pools within both perennial and seasonal streams with clear or moderately turbid water as well as rocky to fine (silt and mud) substrates. The species often favours river sections with emergent aquatic vegetation and woody riparian vegetation.

## Discussion

*Enteromiuspallidus* co-occurs with the chubby head barb, *E.anoplus*, across its distribution range in the CFE. *Enteromiuspallidus* is readily distinguished from *E.anoplus* by possession of two pairs of barbels (*vs* single pair of barbels in *E.anoplus*), fewer lateral scale series (24–31 *vs* 33–37 in *E.anoplus*), presence of irregular scattered spots on the body (*vs* absence in *E.anoplus*). *Enteromiuspallidus* is distinguished from the Amatola barb, *E.amatolicus*, another cyprind minnow that is endemic to the Eastern Cape Province of South Africa, by possession of two pairs of oral barbels (*vs* a single pair in *E.amatolicus*), fewer lateral scale series (24–31 scales *vs* 33–37), fewer scales around the caudal peduncle (12 *vs* 16 scales), and absence of tubercles in mature breeding males (*vs* development of nuptial tubercles in *E.amatolicus* during the breeding season).

Skelton (2001) grouped three southern African smiliogastrins, *E.pallidus*, *E.brevipinnis* and *E.neefi*, into a group which he referred to as the “goldie barb group” based on development of bright golden colour in breeding males. However, the taxonomy, phylogenetic relationships and historical biogeography of this group remain unclear (Engelbrecht and van der Bank, 1996). Studies are required to determine whether the goldie barb group forms a monophyletic unit and shed some light on the diversity and biogeographic patterns of species within this group. There is also need for phylogeographic and ecological studies to assess the mechanisms that shaped the contemporary distribution patterns of *E.pallidus* as it is one of the most widely distributed freshwater fishes in the eastern CFE.

Previous studies have identified sea-level regression, river capture events, inter-drainage dispersal through intermittent freshwater connections and human mediated translocations through construction of inter-basin water transfers as the mechanisms that are likely to have played a role in shaping the distribution and phylogeographic patterns of a number of freshwater fishes in the CFE (Swartz et al. 2007; Chakona and Swartz 2013; Chakona et al. 2015; Cambray and Jubb 1977). However, the evolutionary history for several freshwater fishes in southern Africa, particularly for species within the genus *Enteromius*, remain poorly known. Future studies should aim to use a comparative phylogeographic approach to test whether the genetic structure of freshwater fishes with wide distribution ranges in the CFE, including *E.pallidus*, is congruent with the boundaries of river basins, and determine whether co-distributed species experienced concerted, independent or multiple responses to evolutionary processes.

Recent surveys indicate that *E.pallidus* still persists in at least ten river systems in the eastern CFE including, the Krom, Gamtoos, Baakens, Coega, Swartkops, Sundays, Boesmans, Kariega, Kowie and Great Fish rivers. The species has, however, been affected by a number of human impacts, including hydrological modifications through inter-basin water transfers and excessive water abstraction, pollution, habitat degradation and widespread invasion of the rivers by non-native species (Muller et al. 2015), but its conservation status remains uncertain. Future studies should aim to provide fine scale geographic data and information on the ecology and biology of the species to facilitate effective biodiversity management in the CFE, one of the global endemic hotspots of freshwater fishes.

## Supplementary Material

XML Treatment for Enteromius pallidus
